# Dissimilar Effects of Anagliptin and Sitagliptin on Lipoprotein Subclass in Standard or Strong Statin-Treated Patients with Type-2 Diabetes Mellitus: A Subanalysis of the REASON (Randomized Evaluation of Anagliptin versus Sitagliptin on Low-Density LipoproteiN Cholesterol in Diabetes) Trial

**DOI:** 10.3390/jcm9010093

**Published:** 2019-12-30

**Authors:** Hiroyuki Hirai, Moritake Higa, Takeshi Morimoto, Mio Sakuma, Osamu Arasaki, Takashi Nomiyama, Koichi Node, Shinichiro Ueda, Michio Shimabukuro

**Affiliations:** 1 Department of Diabetes, Endocrinology and Metabolism, Fukushima Medical University, Fukushima 960-1295, Japan; hiroyuki@fmu.ac.jp; 2 Department of Internal Medicine, Shirakawa Kosei General Hospital, Fukushima 961-0005, Japan; 3 Department of Diabetes and Lifestyle-Related Disease Center, Tomishiro Central Hospital, Okinawa 901-0243, Japan; himoritake@yuuai.or.jp; 4 Department of Clinical Epidemiology, Hyogo College of Medicine, Hyogo 663-8501, Japan; morimoto@icekyoto.org (T.M.); mio@hyo-med.ac.jp (M.S.); 5 Department of Cardiology, Tomishiro Central Hospital, Okinawa 901-0243, Japan; oarasaki@yuuai.or.jp; 6 Department of Endocrinology and Diabetes Mellitus, Fukuoka University, Fukuoka 814-0180, Japan; tnomiyama@fukuoka-u.ac.jp; 7 Department of Cardiovascular Medicine, Saga University, Saga 849-8501, Japan; node@med.saga-u.ac.jp; 8 Department of Clinical Pharmacology and Therapeutics, University of the Ryukyus, Okinawa 903-0215, Japan; suedano9@dream.com

**Keywords:** anagliptin, sitagliptin, diabetes mellitus, lipoprotein subclass, statin

## Abstract

The effects of antidiabetic agents on lipoprotein subclasses are assumed to be pivotal, but this assumption has not been studied. We evaluated lipoprotein subclasses in patients, randomly selected from REASON (Randomized Evaluation of Anagliptin versus Sitagliptin On low-density lipoproteiN cholesterol in diabetes) Trial participants, with type-2 diabetes treated with either anagliptin or sitagliptin. We measured total cholesterol (TC) and triglycerides (TG) in 4 (chylomicron (CM), very low-density lipoprotein (VLDL), low density lipoprotein (LDL), and high-density lipoprotein (HDL)) lipoprotein classes and 20 (2 CM, 5 VLDL, 6 LDL, and 7 HDL) lipoprotein subclasses. Between 0 and 52 weeks, TC and TG in lipoprotein and the lipoprotein subclass were distributed differently in patients treated with anagliptin and sitagliptin. The preferable changes in TC and TG levels were observed dominantly in the anagliptin-treated group under standard statin therapy, but the benefits were observed in both the anagliptin- and sitagliptin-treated groups, at least partially under strong statin therapy. In future studies, the atherogenic properties of lipoprotein subclasses might be considered when employing antidiabetic dipeptidyl peptidase-4 (DPP-4) inhibitors, especially in patients with type-2 diabetes who are at risk of atherosclerotic cardiovascular disease (ASCVD) or are undergoing statin treatment.

## 1. Introduction

To prevent atherosclerotic cardiovascular disease (ASCVD) in type-2 diabetes mellitus (T2DM), controlling low-density lipoprotein cholesterol (LDL-C) levels is crucial [1,2]. HMG-CoA reductase inhibitors (statins) are widely used to target recommended levels of LDL-C in T2DM [3,4]. However, at a certain rate, LDL-C target levels are difficult to reach, largely due to statin ineffectiveness and/or intolerance, which limit the potential ASCVD risk reduction of statin treatment [5,6,7]. 

Anagliptin belongs to a class of drugs known as antidiabetic dipeptidyl peptidase-4 (DPP-4) inhibitors. It has been suggested that anagliptin has a unique action among DPP-4 inhibitors due to its ability to lower LDL-C [8]. A Randomized Evaluation of Anagliptin versus Sitagliptin on low-density lipoproteiN cholesterol in diabetes (REASON) Trial was a multicenter, randomized, open-label, parallel-group trial to determine the effectiveness of anagliptin versus sitagliptin on reduction in LDL-C in patients with type-2 diabetes with either prior coronary artery disease (CAD) or CAD risk factors under statin therapy [9]. We found that only anagliptin reduced LDL-C levels, even under treatment with statin [10]. Among the 353 participants in the trial, the frequencies of standard and strong statin users were 278 (78.8%) and 75 (21.2%), respectively [10]. However, the LDL-C lowering effect of anagliptin were not compared between standard and strong statin treatment in the REASON trial.

It has also been shown that the incidence of CAD differs by patterns of lipoprotein subclass [11]. For example, the atherogenic lipoprotein, small dense LDL-C (sdLDL-C), is often observed in patients with T2DM or insulin resistance [12]. Although the effects of antidiabetic agents on the patterns of lipoprotein subclass are assumed to be pivotal, an evaluation testing this assumption has not yet been performed in patients treated with DPP-4 inhibitors, which are among the most frequently prescribed antidiabetic agents [13,14]. 

In this study, we evaluated: (1) the lipoprotein class and subclass patterns of the anagliptin- and sitagliptin-treated groups from REASON Trial participants, and (2) these patterns in strong and standard statin-treated subgroups.

## 2. Materials & Methods

### 2.1. Study Design and Subjects

The REASON Trial was a multicenter, randomized, open-label, active-controlled, parallel-group trial, which was conducted between April 2015 and January 2018 in Japan. The primary end point was the change in LDL-C levels at 52 weeks, and the secondary endpoint was the change in HbA1c levels at 52 weeks. The detailed design of the REASON Trial has been described previously [9,10]. We used an electronic data capture (EDC) system to register and randomize participants and collect data. Randomization to anagliptin or sitagliptin in a 1:1 ratio was performed centrally through the EDC system, with a stochastic minimization algorithm to balance treatment assignment within the strata of center: HbA1c (≥8.0%, <8.0%), use of DPP-4 inhibitors prior to trial registration, sex, BMI (≥25 kg/m^2^, <25 kg/m^2^), and LDL-C (≥130 mg/dL, <130 mg/dL) [9,10]. In this subanalysis of the REASON Trial, 50 participants each from the anagliptin- or sitagliptin-treated groups were randomly selected from the 353 subjects enrolled in the REASON Trial [10]. The Trial and this subanalysis were conducted in accordance with the guidelines established by the Declaration of Helsinki and Japanese clinical study guidelines regarding human subjects in research. The institutional review boards of the University of the Ryukyus (No. 731) and each participating center approved this study, and all patients provided written informed consent prior to participation. This trial was registered at Clinicaltrials.gov on 5 January 2015 (NCT02330406).

### 2.2. Lipoprotein Class and Subclass

We measured the total cholesterol (TC) and triglycerides (TG) of the 4 major lipoprotein classes and 20 lipoprotein subclasses in anagliptin- and sitagliptin-treated groups, and compared the absolute difference between 0 and 52 weeks. Lipoprotein subclasses were measured using gel-permeation high-performance liquid chromatography (GP-HPLC), (LipoSEARCH, Tokyo, Japan) [15,16]. The 4 lipoprotein classes measured included chylomicron (CM), very low-density lipoprotein (VLDL), low-density lipoprotein (LDL), and high-density lipoprotein (HDL). The 20 lipoprotein subclasses measured included 2 CM, 5 VLDL, 6 LDL, and 7 HDL subclasses, according to prior classification (Table 1) [15,16]. We also analyzed changes in lipoprotein subclass in the strong or standard statin-treated subgroups.

### 2.3. Clinical Variables

Clinical variables included age, sex, height, body weight (BW), systolic blood pressure (SBP), diastolic blood pressure (DBP), and heart rate (HR). Body mass index (BMI) was calculated as BW (kg)/height (m)^2^. Medication records were accessed, using an electronic data capture (EDC) system, for prior use of DPP-4 inhibitors, sulfonylureas, glinides, metformin, pioglitazone, α-glucosidase inhibitors, sodium-glucose cotransporter inhibitors, insulin, and lipid-lowering medications (statins, ezetimibe, fibrates, eicosapentaenoic acid, and nicotinic acid). Statins were classified either as standard (pravastatin, simvastatin and fluvastatin) or strong (atorvastatin, rosuvastatin, and pitavastatin) [17]. Biochemical tests included fasting plasma glucose (FPG), HbA1c, TC, TG, HDL-C, aspartate transaminase (AST), alanine transaminase (ALT), blood urea nitrogen (BUN), and serum creatinine. Estimated glomerular filtration rate (e-GFR) was calculated using the Japanese formula for GFR estimation: eGFR (mL/min/1.73 m^2^) = 194 × serum creatinine (mg/dL)^−1.094^ × age (years)^−0.287^ [18].

### 2.4. Statistical Analysis

Statistical analysis was performed as previously described [9,10]. In brief, categorical variables are shown as number and percentage, and continuous variables as mean and standard derivation or median and quartile, if appropriate. Comparisons between weeks 0 and 52 were analyzed by paired *t*-test, and comparisons between the anagliptin and sitagliptin groups were by *t*-test. Because the allocation of anagliptin and sitagliptin was conducted at random, no adjustment for comparisons between groups was done. In addition, the changes over time and differences between groups were clinically defined, and adjustment for the multiplicity of *p*-value was not considered. All Statistical analyses were conducted using JMP 13.1 (SAS Institute Inc., Cary, NC, USA) and SAS 9.4 (SAS Institute Inc.). Two-tailed *p*-values < 0.05 were considered statistically significant.

## 3. Results

### 3.1. Characteristics of Subjects

Baseline characteristics are shown in Table 2. Participants (*n* = 100; 50 each of anagliptin- and sitagliptin-treated groups) with a mean age of 68 (9) years, 57% male, BMI of 26.8 (3.78), HbA1c of 7.0% (0.7) and LDL-C of 113 (24) mg/dL were comparable with the REASON Trial participants (*n* = 353). Laboratory values, and the proportions of antidiabetic and lipid-lowing drugs, were comparable in both groups.

### 3.2. TC and TG in Lipoprotein Classes and Subclasses 

Values of TC and TG at 0 and 52 weeks in the 4 lipoprotein classes and 20 lipoprotein subclasses studied are shown in Supplementary Tables S1 and S2. Differences between 0 and 52 weeks in TC and TG in CM, VLDL, LDL, and HDL, and those of the 20 lipoprotein subclasses studied, are shown in Table 1. In the 4 classes studied, TC in LDL was decreased in the anagliptin group and TC in CM was increased in the sitagliptin group. In the 20 subclasses, 5 of the 6 subclasses of LDL were decreased in the anagliptin group, while 2 of these were decreased in the sitagliptin group. Within the 7 HDL subclasses, TC increased or tended to be increased in P14–P17 in both groups, while TC decreased in P18–19 only in the anagliptin group. TG was increased in 4 out of 7 HDL subclasses only in the sitagliptin group.

### 3.3. TC and TG in Lipoprotein Classes and Subclasses in Standard or Strong Statin Subgroups

Additionally, we analyzed lipoprotein classes and subclasses in patients treated with strong or standard statins. Frequencies of standard and strong statin users were comparable between the anagliptin and sitagliptin groups (Table 2). In the standard statin subgroup (Table 3), TC in LDL, TC of 5 LDL subclasses, and TC of 2 HDL subclasses were decreased only among the anagliptin group. Additionally, TG in VLDL and TG of 3 VLDL subclasses were decreased only in the anagliptin group. TG in HDL was increased in anagliptin group. In the strong statin subgroup (Table 4), TC in LDL and LDL in the small LDL subclass were decreased significantly in the anagliptin group, and tended to be decreased in the sitagliptin group. TG in VLDL and TG in the 5 VLDL subclasses were decreased in both the anagliptin and sitagliptin groups.

## 4. Discussion

In this study, we measured changes in TC and TG from 0 to 52 weeks in lipoprotein classes and subclasses in anagliptin- and sitagliptin-treated patients with type-2 diabetes who had concomitantly taken either strong or standard statins. We obtained two major findings from this analysis, which add to those of the prior REASON trial. First, we found that TC in lipoprotein classes and subclasses was altered at 52 weeks in both the anagliptin- and sitagliptin-treated groups. Specifically, TC in LDL was decreased only in anagliptin-treated patients, and TC in CM was increased only in patients treated with sitagliptin. However, TG in VLDL and VLDL subclasses were decreased similarly in both groups. Second, differences in TC in LDL and LDL subclasses, and TG in VLDL and VLDL subclasses, were observed both in the standard and strong statin subgroups of anagliptin- and sitagliptin-treated patients. However, the changes in TC and TG levels in lipoprotein classes and subclasses were distributed differently between the anagliptin- and sitagliptin-treated groups. The preferable changes in TC and TG levels (lowering TC in LDL and lowering TG in VLDL) were observed dominantly in anagliptin-treated group under standard statin therapy, but the beneficial effects were observed both in anagliptin- and sitagliptin-treated group under strong statin therapy.

Among the 20 lipoprotein subclasses studied, sdLDL-C is considered highly atherogenic [11]. Additionally, sdLDL-C is believed to reflect an increase in remnants or TG-rich lipoproteins, and is often observed in patients with T2DM or insulin resistance [12]. Although sdLDL-C and other lipoproteins are possible targets in treating ASCVD [12], previous studies have not fully studied the effects of antidiabetic drugs, including DPP-4 inhibitors, on the lipoprotein profiles. We found, for the first time, that anagliptin decreased TC in sdLDL, in addition to the known effect of lowering LDL cholesterol [10]. Previous in vitro and in vivo studies may provide insights into the mechanisms underlying the lowering of LDL cholesterol by anagliptin. Yano et al. reported that anagliptin decreased LDL cholesterol by downregulating synthesis of TC via liver sterol regulatory element-binding protein-2 (SREBP-2) [8]. Additionally, Goto et al. reported that anagliptin can decrease TC absorption from the small intestine [19]. In addition to lowering TC in LDL, the current study also showed that anagliptin had the unique effect of decreasing TC in small and very small HDL subclasses. This effect coincided with changes in TG levels in VLDL subclasses, which was also observed with sitagliptin treatment. Although the atherogenicity of HDL subclasses are still a matter of controversy, the changes observed here in HDL subclasses may play a functional role [20]. 

Although statins are beneficial in preventing ASCVD [3,4], residual risk after statin treatment should be considered in specific conditions [5,6,12,21,22,23,24,25,26]. In T2DM, possible candidates for residual risks include sdLDL [12], postprandial hypertriglyceridemia [25], and TG-rich lipoproteins [21,25,26]. Our findings may provide a unique approach to this important “beyond statin” issue [21,22,23,24]. Previous studies have reported the effects of anagliptin on the lipid profiles of patients with type-2 diabetes, but these were not controlled for statin treatment [14,19,27]. We, for the first time, evaluated the effects of DPP-4 inhibitors on lipid and lipoprotein profiles in patients with type-2 diabetes undergoing statin treatment. Further, this study demonstrated the differing impacts of anagliptin and sitagliptin on TC in LDL and LDL subclasses, and on TG in VLDL subclasses in patients undergoing treatment with standard statins. However, beneficial effects were observed both in anagliptin- and sitagliptin-treated group under strong statin therapy. We could not explain the differences in effect of two DPP4 inhibitors under standard- and strong-statin therapy from our results.

At baseline, the mean (SD) LDL cholesterol was 126 (25) mg/dL in the standard statin group and 110 (23) mg/dL in the strong statin group. In the Japan Atherosclerosis Society (JAS) Guidelines for Prevention of Atherosclerotic Cardiovascular Diseases 2017, it is recommended that patients with diabetes maintain a LDL cholesterol level below 120 mg/dL [3]. Therefore, we cannot determine exactly why standard statins could not be switched to strong statins in these patients, but we might reasonably assume the reason is statin intolerance, i.e., the status in which an adequate dose of statin cannot be continuously used because of adverse effects [5,6]. The major reasons for statin intolerance have been reported to be statin-associated muscle symptoms (SAMS) and increased CK levels and their temporal association with initiation of statin therapy [28], partially due to solute carrier organic anion transporter family member 1B1 (SLCO1B1) variant alleles [29]. Nagar et al. reported that statin intolerance was observed in approximately 10% in Japanese patients with high CAD risk [5]. Although we cannot estimate any clinical benefit from the present results, it might be interesting to observe long-term effects of anagliptin in patients with T2DM with intolerance to strong statins.

Our study has several limitations. First, the sample size was small, especially in the standard and strong statin subgroups. However, there is a clear difference between anagliptin- and sitagliptin-treated groups, especially among the standard statin subgroup. The degree of the difference in LDL-C levels is quite minimal between anagliptin and sitagliptin groups, and thus, one should be careful when estimating its clinical utility. This study evaluated lipoprotein and the lipoprotein subclass by the largest number of diabetic patients treated with DPP-4 inhibitors so far, and therefore, should be reliable. Further studies may be warranted to confirm these results. Second, the participants were all Japanese, and, therefore, our observations should be re-evaluated in other races. Third, because the study period was 52 weeks, we could not evaluate the onset of ASCVD events. Although clinical trials on ASCVD events in patients treated with DPP-4 inhibitors have been reported [30,31], the effects of this class of antidiabetic drugs should be considered, especially on diabetic dyslipidemia.

## 5. Conclusions

Changes in TC and TG levels in lipoprotein classes and subclasses were distributed differently between patients treated with anagliptin and sitagliptin. The preferable changes in TC and TG levels were observed dominantly in the anagliptin-treated group under standard statin therapy, but the benefits were observed both in anagliptin- and sitagliptin-treated group, at least partially, under strong statin therapy. In future studies, the atherogenic properties of lipoprotein subclasses might be considered when employing antidiabetic DPP-4 inhibitors, especially in patients with type-2 diabetes who are at risk for ASCVD or who are undergoing statin treatment.

## Figures and Tables

**Table 1:** General characteristics.

Variables	All	Anagliptin	Sitagliptin
*N*	100	50	50
Age (years)	68 (9)	68 (9)	68 (9)
Sex (Men, %)	57 (57)	30 (60)	27 (54)
Height (cm)	158.7 (8.9)	158.9 (8.8)	158.5 (9.1)
Body weight (kg)	67.9 (12.6)	68.4 (12.6)	67.3 (12.7)
BMI (kg/m^2^)	26.8 (3.8)	27.0 (3.7)	26.7 (3.9)
SBP (mmHg)	134 (16)	137 (17)	132 (15)
DBP (mmHg)	72 (12)	75 (12)	70 (12)
Heart Rate (beats/min)	75 (12)	78 (12)	71 (12)
TC (mg/dL)	191 (29)	192 (28)	190 (31)
HDL-C (mg/dL)	54 (13)	54 (14)	54 (13)
Triglyceride (mg/dL)	133 (87, 177)	144 (104, 189)	118 (81, 165)
LDL-C (mg/dL)	113 (24)	111 (22)	114 (26)
FPG (mg/dL)	140 (35)	147 (41)	133 (26)
HbA1c (%)	7.0 (0.7)	7.1 (0.8)	6.9 (0.6)
AST (IU/L)	25 (18)	28 (24)	23 (10)
ALT (IU/L)	25 (17)	28 (20)	22 (11)
Creatinine (mg/dL)	0.83 (0.24)	0.83 (0.22)	0.83 (0.27)
eGFR (mL/min/1.73 m^2^)	68.0 (18.1)	67.6 (17.1)	68.5 (19.3)
DPP-4 inhibitors previously used (%)	75 (75)	37 (74)	38 (76)
Antidiabetic drugs (%)	70 (70)	36 (72)	34 (68)
Metformin (%)	47 (47)	24 (48)	23 (46)
Thiazolidinedione (%)	17 (17)	5 (10)	12 (24)
Sulfonylureas (%)	29 (29)	15 (30)	14 (28)
α-glucosidase inhibitor (%)	22 (22)	8 (16)	14 (28)
Glinide (%)	4 (4)	3 (6)	1 (2)
Insulin (%)	8 (8)	5 (10)	3 (6)
sodium-glucose cotransporter 2 inhibitors (%)	17 (17)	8 (16)	9 (18)
Lipid lowering agents			
Statin (%)	100 (100)	50 (100)	50 (100)
Standard statin or Strong statin (%)	19 (19) vs. 81 (81)	9 (18) vs. 41 (82)	10 (20) vs. 40 (80)
Ezetimib (%)	8 (8)	5 (10)	3 (6)
Fibrate (%)	5 (5)	2 (4)	3 (6)
EPA or EPA + DHA (%)	11 (11)	6 (12)	5 (10)

Mean (SD), *n* (%) or median [IQC 25%, 75%].

**Table 2:** Differences in total cholesterol and triglycerides in lipoprotein classes and subclasses in patients treated either with anagliptin or sitagliptin. All subjects.

	Anagliptin	Sitagliptin	*P*	Class	Anagliptin	Sitagliptin	Peak Number of 20 Subclass	Subclass Name	Anagliptin	Sitagliptin
TC	−5.99 (23.2)	−0.66 (36.0)	0.38	CM	0.75 (3.8)	1.52 (3.8) **	P01	CM	0.51 (2.9)	1.11 (2.7) **
							P02	CM	0.24 (1.0)	0.41 (1.1) *
				VLDL	−1.58 (9.7)	−0.75 (10.3)	P03	large VLDL	0.24 (1.7)	0.38 (1.9)
							P04	large VLDL	−0.22 (2.3)	0.26 (2.4)
							P05	large VLDL	−0.83 (4.2)	−0.80 (4.5)
							P06	medium VLDL	0.10 (2.3)	0.19 (2.4)
							P07	small VLDL	−0.87 (1.5) **	−0.78 (1.8) **
				LDL	−5.70 (11.5) **	−4.02 (19.4)	P08	large LDL	−1.18 (4.5)	−0.59 (6.3)
							P09	medium LDL	−2.68 (5.8) **	−2.18 (9.7)
							P10	small LDL	−1.29 (3.3) **	−0.91 (4.3)
							P11	very small LDL	−0.65 (1.0) **	−0.60 (1.2) **
							P12	very small LDL	0.19 (0.3) **	0.28 (0.3) **
							P13	very small LDL	−0.08 (0.2) **	−0.03 (0.2)
				HDL	0.53 (6.3)	2.58 (10.3)	P14	very large HDL	0.13 (0.3) **	0.19 (0.2) **
							P15	very large HDL	0.03 (1.1)	0.31 (0.9) *
							P16	large HDL	0.56 (3.1)	1.54 (3.4) *
							P17	medium HDL	0.89 (2.9) *	1.07 (4.1)
							P18	small HDL	−0.76 (1.9) **	−0.48 (2.5)
							P19	very small HDL	−0.31 (0.7) **	−0.10 (0.7)
							P20	very small HDL	0.00 (0.2)	0.06 (0.2)
TG	−31.45 (60.6) **	−18.27 (57.9) *	0.27	CM	−2.16 (19.4)	0.92 (16.8)	P01	CM	−0.76 (14.1)	1.37 (11.9)
							P02	CM	−1.40 (5.4)	−0.45 (5.1)
				VLDL	−28.3 (35.8) **	−20.2 (35.5) **	P03	large VLDL	−4.54 (8.6) **	−2.90 (8.8) *
							P04	large VLDL	−9.61 (12.4) **	−6.80 (12.7) **
							P05	large VLDL	−9.52 (10.9) **	−6.90 (10.4) **
							P06	medium VLDL	−3.42 (4.4) **	−2.40 (4.0) **
							P07	small VLDL	−1.17 (1.3) **	−1.19 (1.6) **
				LDL	−1.04 (5.1)	−0.40 (6.4)	P08	large LDL	−0.79 (1.7) **	−0.76 (2.5) *
							P09	medium LDL	−0.27 (1.9)	−0.14 (2.4)
							P10	small LDL	0.04 (1.2)	0.26 (1.2)
							P11	very small LDL	−0.09 (0.4)	0.00 (0.4)
							P12	very small LDL	0.08 (0.3)	0.17 (0.3) **
							P13	very small LDL	−0.01 (0.2)	0.06 (0.2)
				HDL	0.00 (5.2)	1.41 (6.4)	P14	very large HDL	0.06 (0.2) *	0.12 (0.3) **
							P15	very large HDL	−0.09 (0.6)	0.18 (0.6) *
							P16	large HDL	−0.02 (1.7)	0.58 (1.9) *
							P17	medium HDL	0.16 (1.8)	0.37 (2.3)
							P18	small HDL	−0.14 (0.9)	0.00 (1.1)
							P19	very small HDL	−0.03 (0.3)	0.05 (0.3)
							P20	very small HDL	0.06 (0.3)	0.11 (0.3) *

* *P* < 0.05, ** *P* < 0.01 between week 0 and 52. TC: total cholesterol, TG: triglycerides, CM: chylomicron, VLDL: very low-density lipoprotein, LDL: low-density lipoprotein, HDL: high-density lipoprotein. Mean (SD), *n* (%).

**Table 3:** Differences in total cholesterol and triglycerides in lipoprotein classes and subclasses in patients treated either with anagliptin or sitagliptin. Standard statin group.

	Anagliptin	Sitagliptin	*P*	Class	Anagliptin	Sitagliptin	Peak Number of 20 Subclass	Subclass Name	Anagliptin	Sitagliptin
TC	−17.10 (23.6)	13.84 (53.5)	0.13	CM	2.26 (2.9) *	1.73 (3.4)	P01	CM	1.65 (2.0) *	1.15 (2.3)
							P02	CM	0.61 (0.9)	0.58 (1.1)
				VLDL	−4.48 (12.2)	3.30 (11.0)	P03	large VLDL	0.54 (1.9)	0.95 (1.9)
							P04	large VLDL	−0.17 (2.7)	1.14 (2.8)
							P05	large VLDL	−2.88 (5.4)	0.53 (4.7)
							P06	medium VLDL	−0.53 (2.1)	0.74 (2.8)
							P07	small VLDL	−1.43 (1.7) *	−0.06 (2.3)
				LDL	−12.40 (13.0) *	0.89 (27.9)	P08	large LDL	−4.09 (4.7) *	1.18 (9.1)
							P09	medium LDL	−6.01 (6.7) *	−0.41 (13.7)
							P10	small LDL	−1.59 (1.8) *	0.09 (4.7)
							P11	very small LDL	−0.84 (0.9) *	−0.48 (1.4)
							P12	very small LDL	0.28 (0.4)	0.45 (0.3) **
							P13	very small LDL	−0.12 (0.1) *	0.05 (0.3)
				HDL	−2.50 (4.5)	7.92 (15.2)	P14	very large HDL	0.11 (0.1) *	0.33 (0.3) **
							P15	very large HDL	−0.11 (0.2)	0.93 (1.0) *
							P16	large HDL	−0.41 (2.5)	2.94 (4.8)
							P17	medium HDL	0.37 (1.4)	2.57 (5.8)
							P18	small HDL	−1.74 (1.7) *	0.77 (3.3)
							P19	very small HDL	−0.66 (0.6) **	0.20 (0.9)
							P20	very small HDL	−0.08 (0.1)	0.19 (0.3)
TG	−10.69 (36.4)	−1.88 (63.5)	0.72	CM	6.20 (13.0)	2.15 (13.9)	P01	CM	5.13 (9.5)	1.96 (9.1)
							P02	CM	1.07 (3.7)	0.19 (4.9)
				VLDL	−19.3 (24.6) *	−9.03 (41.6)	P03	large VLDL	−1.00 (6.2)	−0.87 (9.5)
							P04	large VLDL	−5.86 (9.6)	−3.70 (15.4)
							P05	large VLDL	−7.95 (8.3) *	−3.27 (12.4)
							P06	medium VLDL	−3.20 (3.5) *	−0.77 (4.5)
							P07	small VLDL	−1.26 (1.2) *	−0.42 (2.1)
				LDL	−0.31 (3.8)	2.08 (6.3)	P08	large LDL	−0.89 (1.4)	0.46 (2.8)
							P09	medium LDL	−0.09 (1.5)	0.74 (2.4)
							P10	small LDL	0.35 (1.0)	0.58 (1.0)
							P11	very small LDL	0.03 (0.3)	0.03 (0.4)
							P12	very small LDL	0.23 (0.3) *	0.20 (0.2) *
							P13	very small LDL	0.07 (0.1)	0.06 (0.2)
				HDL	2.68 (3.2) *	2.93 (5.9)	P14	very large HDL	0.15 (0.2) *	0.15 (0.2) *
							P15	very large HDL	0.13 (0.2)	0.32 (0.4) *
							P16	large HDL	0.64 (0.9)	0.98 (1.9)
							P17	medium HDL	1.20 (1.2) *	0.83 (2.1)
							P18	small HDL	0.30 (0.6)	0.35 (1.1)
							P19	very small HDL	0.13 (0.2)	0.11 (0.3)
							P20	very small HDL	0.14 (0.2)	0.19 (0.3) *

* *P* < 0.05, ** *P* < 0.01 between week 0 and 52. TC: total cholesterol, TG: triglycerides, CM: chylomicron, VLDL: very low-density lipoprotein, LDL: low-density lipoprotein, HDL: high-density lipoprotein. Mean (SD), *n* (%).

**Table 4:** Differences in total cholesterol and triglycerides in lipoprotein classes and subclasses in patients treated either with anagliptin or sitagliptin. Strong statin group.

	Anagliptin	Sitagliptin	*P*	Class	Anagliptin	Sitagliptin	Peak Number of 20 Subclass	Subclass Name	Anagliptin	Sitagliptin
TC	−3.55 (22.7)	−4.29 (29.9)	0.90	CM	0.42 (3.9)	1.47 (4.0) *	P01	CM	0.26 (3.0)	1.10 (2.9) *
							P02	CM	0.16 (1.0)	0.37 (1.1) *
				VLDL	−0.95 (9.2)	−1.76 (10.0)	P03	large VLDL	0.17 (1.6)	0.24 (1.9)
							P04	large VLDL	−0.23 (2.3)	0.04 (2.3)
							P05	large VLDL	−0.37 (3.9)	−1.13 (4.5)
							P06	medium VLDL	0.24 (2.3)	0.05 (2.3)
							P07	small VLDL	−0.75 (1.4) **	−0.96 (1.6) **
				LDL	−4.22 (10.8) *	−5.24 (16.9)	P08	large LDL	−0.54 (4.2)	−1.03 (5.5)
							P09	medium LDL	−1.95 (5.4) *	−2.62 (8.5)
							P10	small LDL	−1.23 (3.5) *	−1.15 (4.2)
							P11	very small LDL	−0.60 (1.0) **	−0.63 (1.1) **
							P12	very small LDL	0.17 (0.3) **	0.24 (0.3) **
							P13	very small LDL	−0.07 (0.2) *	−0.05 (0.2)
				HDL	1.20 (6.5)	1.24 (8.5)	P14	very large HDL	0.13 (0.3) **	0.15 (0.2) **
							P15	very large HDL	0.06 (1.2)	0.16 (0.8)
							P16	large HDL	0.77 (3.2)	1.19 (2.9) *
							P17	medium HDL	1.00 (3.1) *	0.69 (3.5)
							P18	small HDL	−0.55 (1.9)	−0.79 (2.2) *
							P19	very small HDL	−0.24 (0.7) *	−0.18 (0.7)
							P20	very small HDL	0.02 (0.2)	0.03 (0.2)
TG	−36.01 (64.2) **	−22.36 (56.5) *	0.31	CM	−3.99 (20.1)	0.62 (17.6)	P01	CM	−2.05 (14.7)	1.23 (12.6)
							P02	CM	−1.94 (5.6) *	−0.61 (5.2)
				VLDL	−30.23 (37.8) **	−22.99 (33.8) **	P03	large VLDL	−5.32 (8.9) **	−3.41 (8.6) *
							P04	large VLDL	−10.44 (12.9) **	−7.57 (12.1) **
							P05	large VLDL	−9.86 (11.4) **	−7.81 (9.8) **
							P06	medium VLDL	−3.47 (4.6) **	−2.81 (3.8) **
							P07	small VLDL	−1.15 (1.3) **	−1.39 (1.5) **
				LDL	−1.20 (5.4)	−1.02 (6.3)	P08	large LDL	−0.77 (1.8) *	−1.06 (2.4) *
							P09	medium LDL	−0.31 (2.0)	−0.36 (2.4)
							P10	small LDL	−0.03 (1.2)	0.18 (1.2)
							P11	very small LDL	−0.11 (0.4)	−0.01 (0.4)
							P12	very small LDL	0.05 (0.3)	0.17 (0.4) *
							P13	very small LDL	−0.03 (0.2)	0.05 (0.2)
				HDL	−0.58 (5.4)	1.03 (6.5)	P14	very large HDL	0.04 (0.2)	0.12 (0.3) *
							P15	very large HDL	−0.13 (0.7)	0.15 (0.6)
							P16	large HDL	−0.16 (1.7)	0.48 (1.9)
							P17	medium HDL	−0.07 (1.8)	0.25 (2.4)
							P18	small HDL	−0.23 (1.0)	−0.08 (1.1)
							P19	very small HDL	−0.07 (0.3)	0.03 (0.3)
							P20	very small HDL	0.04 (0.3)	0.09 (0.3)

* *P* < 0.05, ** *P* < 0.01 between week 0 and 52. TC: total cholesterol, TG: triglycerides, CM: chylomicron, VLDL: very low-density lipoprotein, LDL: low-density lipoprotein, HDL: high-density lipoprotein. Mean (SD), *n* (%).

## Supplementary Materials

The following are available online at https://www.mdpi.com/2077-0383/9/1/93/s1. Table S1: Values of total cholesterol in lipoprotein and lipoprotein subclass in patients treated either with anagliptin or sitagliptin at 0 and 52 week, Table S2: Values of triglycerides in lipoprotein and lipoprotein subclass in patients treated either with anagliptin or sitagliptin at 0 and 52 week.

## References

[1] Grundy S.M., Stone N.J., Bailey A.L., Beam C., Birtcher K.K., Blumenthal R.S., Braun L.T., de Ferranti S., Faiella-Tommasino J., Forman D.E. (2019). 2018 AHA/ACC/AACVPR/AAPA/ABC/ACPM/ADA/AGS/APhA/ASPC/NLA/PCNA Guideline on the Management of Blood Cholesterol: Executive Summary: A Report of the American College of Cardiology/American Heart Association Task Force on Clinical Practice Guidelines. J. Am. Coll. Cardiol..

[2] Cosentino F., Grant P.J., Aboyans V., Bailey C.J., Ceriello A., Delgado V., Federici M., Filippatos G., Grobbee D.E., Hansen T.B. (2019). 2019 ESC Guidelines on diabetes, pre-diabetes, and cardiovascular diseases developed in collaboration with the EASD. Eur. Heart J..

[3] Kinoshita M., Yokote K., Arai H., Iida M., Ishigaki Y., Ishibashi S., Umemoto S., Egusa G., Ohmura H., Okamura T. (2018). Japan Atherosclerosis Society (JAS) guidelines for prevention of atherosclerotic cardiovascular diseases 2017. J. Atheroscler. Thromb..

[4] Arnett D.K., Blumenthal R.S., Albert M.A., Buroker A.B., Goldberger Z.D., Hahn E.J., Himmelfarb C.D., Khera A., Lloyd-Jones D., McEvoy J.W. (2019). 2019 ACC/AHA guideline on the primary prevention of cardiovascular disease: Executive summary: A report of the American College of Cardiology/American Heart Association Task Force on clinical practice guidelines. Circulation.

[5] Nagar S.P., Rane P.P., Fox K.M., Meyers J., Davis K., Beaubrun A., Inomata H., Qian Y., Kajinami K. (2018). Treatment patterns, statin intolerance, and subsequent cardiovascular events among Japanese patients with high cardiovascular risk initiating statin therapy. Circ. J..

[6] Toth P.P., Patti A.M., Giglio R.V., Nikolic D., Castellino G., Rizzo M., Banach M. (2018). Management of statin intolerance in 2018: Still more questions than answers. Am. J. Cardiovasc. Drugs.

[7] Rodriguez V., Newman J.D., Schwartzbard A.Z. (2018). Towards more specific treatment for diabetic dyslipidemia. Curr. Opin. Lipidol..

[8] Yano W., Inoue N., Ito S., Itou T., Yasumura M., Yoshinaka Y., Hagita S., Goto M., Nakagawa T., Inoue K. (2017). Mechanism of lipid-lowering action of the dipeptidyl peptidase-4 inhibitor, anagliptin, in low-density lipoprotein receptor-deficient mice. J. Diabetes Investig..

[9] Ueda S., Shimabukuro M., Arasaki O., Node K., Nomiyama T., Morimoto T. (2018). Effect of anagliptin and sitagliptin on low-density lipoprotein cholesterol in type 2 diabetic patients with dyslipidemia and cardiovascular risk: Rationale and study design of the REASON trial. Cardiovasc. Drugs Ther..

[10] Morimoto T., Sakuma I., Sakuma M., Tokushige A., Natsuaki M., Asahi T., Shimabukuro M., Nomiyama T., Arasaki O., Node K. (2019). Randomized evaluation of anagliptin vs sitagliptin on low-density lipoprotein cholesterol in diabetes (REASON) trial: A 52-week, open-label, randomized clinical trial. Sci. Rep..

[11] Krauss R.M. (2004). Lipids and lipoproteins in patients with type 2 diabetes. Diabetes Care.

[12] Hirano T. (2018). Pathophysiology of diabetic dyslipidemia. J. Atheroscler. Thromb..

[13] Masuda D., Kobayashi T., Sairyou M., Hanada H., Ohama T., Koseki M., Nishida M., Maeda N., Kihara S., Minami T. (2018). Effects of a dipeptidyl peptidase 4 inhibitor sitagliptin on glycemic control and lipoprotein metabolism in patients with type 2 diabetes mellitus (GLORIA trial). J. Atheroscler. Thromb..

[14] Kurozumi A., Okada Y., Arao T., Kobayashi T., Masuda D., Yamashita S., Tanaka Y. (2018). Comparison of effects of anagliptin and alogliptin on serum lipid profile in type 2 diabetes mellitus patients. J. Diabetes Investig..

[15] Okazaki M., Usui S., Ishigami M., Sakai N., Nakamura T., Matsuzawa Y., Yamashita S. (2005). Identification of unique lipoprotein subclasses for visceral obesity by component analysis of cholesterol profile in high-performance liquid chromatography. Arterioscler. Thromb. Vasc. Biol..

[16] Toshima G.I.Y., Kimura F., Matsumoto Y., Miura M., Takahashi J., Yasuda H., Arai N., Mizutani H., Hata K., Usui S. (2013). LipoSEARCH; analytical GP-HPLC method for lipoprotein profiling and its applications. J. Biol. Macromol..

[17] Natsuaki M., Furukawa Y., Morimoto T., Nakagawa Y., Ono K., Kaburagi S., Inada T., Mitsuoka H., Taniguchi R., Nakano A. (2012). Intensity of statin therapy, achieved low-density lipoprotein cholesterol levels and cardiovascular outcomes in Japanese patients after coronary revascularization. Perspectives from the CREDO-Kyoto registry cohort-2. Circ. J..

[18] Matsuo S., Imai E., Horio M., Yasuda Y., Tomita K., Nitta K., Yamagata K., Tomino Y., Yokoyama H., Hishida A. (2009). Revised equations for estimated GFR from serum creatinine in Japan. Am. J. Kidney Dis..

[19] Goto M., Furuta S., Yamashita S., Hashimoto H., Yano W., Inoue N., Kato N., Kaku K. (2018). Dipeptidyl peptidase 4 inhibitor anagliptin ameliorates hypercholesterolemia in hypercholesterolemic mice through inhibition of intestinal cholesterol transport. J. Diabetes Investig..

[20] Zhang Y., Gordon S.M., Xi H., Choi S., Paz M.A., Sun R., Yang W., Saredy J., Khan M., Remaley A.T. (2019). HDL subclass proteomic analysis and functional implication of protein dynamic change during HDL maturation. Redox Biol..

[21] Lawler P.R., Akinkuolie A.O., Harada P., Glynn R.J., Chasman D.I., Ridker P.M., Mora S. (2017). Residual risk of atherosclerotic cardiovascular events in relation to reductions in very-low-density lipoproteins. J. Am. Heart Assoc..

[22] Lloyd-Jones D.M., Morris P.B., Ballantyne C.M., Birtcher K.K., Daly D.D., DePalma S.M., Minissian M.B., Orringer C.E., Smith S.C. (2017). 2017 focused update of the 2016 ACC expert consensus decision pathway on the role of non-statin therapies for ldl-cholesterol lowering in the management of atherosclerotic cardiovascular disease risk: A report of the American College of Cardiology task force on expert consensus decision pathways. J. Am. Coll. Cardiol..

[23] Ganda O.P., Bhatt D.L., Mason R.P., Miller M., Boden W.E. (2018). Unmet need for adjunctive dyslipidemia therapy in hypertriglyceridemia management. J. Am. Coll. Cardiol..

[24] Fruchart J.C., Santos R.D., Aguilar-Salinas C., Aikawa M., Al Rasadi K., Amarenco P., Barter P.J., Ceska R., Corsini A., Despres J.P. (2019). The selective peroxisome proliferator-activated receptor alpha modulator (SPPARMalpha) paradigm: Conceptual framework and therapeutic potential: A consensus statement from the International Atherosclerosis Society (IAS) and the Residual Risk Reduction Initiative (R3i) Foundation. Cardiovasc. Diabetol..

[25] Sandesara P.B., Virani S.S., Fazio S., Shapiro M.D. (2019). The forgotten lipids: Triglycerides, remnant cholesterol, and atherosclerotic cardiovascular disease risk. Endocr. Rev..

[26] Andersen I.R., Sondergaard E., Sorensen L.P., Nellemann B., Gormsen L.C., Jensen M.D., Nielsen S. (2017). Increased VLDL-TG fatty acid storage in skeletal muscle in men with type 2 diabetes. J. Clin. Endocrinol. Metabol..

[27] Chiba Y., Yamakawa T., Tsuchiya H., Oba M., Suzuki D., Danno H., Takatsuka Y., Shigematsu H., Kaneshiro M., Terauchi Y. (2018). Effect of anagliptin on glycemic and lipid profile in patients with type 2 diabetes mellitus. J. Clin. Med. Res..

[28] Stroes E.S., Thompson P.D., Corsini A., Vladutiu G.D., Raal F.J., Ray K.K., Roden M., Stein E., Tokgozoglu L., Nordestgaard B.G. (2015). Statin-associated muscle symptoms: Impact on statin therapy-European Atherosclerosis Society Consensus Panel Statement on Assessment, Aetiology and Management. Eur. Heart J..

[29] Arrigoni E., Del Re M., Fidilio L., Fogli S., Danesi R., Di Paolo A. (2017). Pharmacogenetic foundations of therapeutic efficacy and adverse events of statins. Int. J. Mol. Sci..

[30] Nauck M.A., Meier J.J., Cavender M.A., Abd El Aziz M., Drucker D.J. (2017). Cardiovascular actions and clinical outcomes with glucagon-like peptide-1 receptor agonists and dipeptidyl peptidase-4 inhibitors. Circulation.

[31] Rosenstock J., Perkovic V., Johansen O.E., Cooper M.E., Kahn S.E., Marx N., Alexander J.H., Pencina M., Toto R.D., Wanner C. (2019). Effect of linagliptin vs placebo on major cardiovascular events in adults with type 2 diabetes and high cardiovascular and renal risk: The CARMELINA randomized clinical trial. JAMA.

