# Supplementary material for: Dissimilar Effects of Anagliptin and Sitagliptin on Lipoprotein Subclass in Standard or Strong Statin-Treated Patients with Type-2 Diabetes Mellitus: A Subanalysis of the REASON (Randomized Evaluation of Anagliptin versus Sitagliptin on Low-Density LipoproteiN Cholesterol in Diabetes) Trial
Source: J Clin Med. 2019 Dec 30;9(1):93. doi: 10.3390/jcm9010093 (PMC7019317; doi:10.3390/jcm9010093)
Supplement: Supplementary file 1 [file jcm-09-00093-s001.zip › jcm-657904-supplementary/Hirai_Supplement_1.pdf]

**Supplement 1. Values of total cholesterol in lipoprotein and lipoprotein subclass in patients treated either with anagliptin or sitagliptin at 0 and 52 week**

**0 week**

| | Anagliptin | Sitagliptin | P | 4 subclass | Anagliptin | Sitagliptin | Peak number of 20 subclass | Subclass name | Anagliptin | Sitagliptin |
|---|---|---|---|---|---|---|---|---|---|---|
| TC | 180.2 [164.7 , 203.2] | 186.78 [162.0 , 206.4] | 0.89 | CM | 3.89 [1.6 , 6.2] | 2.73 [1.2 , 4.9] | P01 | CM | 2.30 [0.9 , 3.8] | 1.48 [0.7 , 3.0] |
| | | | | | | | P02 | CM | 1.59 [0.8 , 2.5] | 1.22 [0.6 , 2.1] |
| | | | | VLDL | 41.77 [32.5 , 53.0] | 41.55 [32.9 , 51.5] | P03 | large VLDL | 4.27 [2.5 , 5.8] | 3.70 [2.2 , 5.2] |
| | | | | | | | P04 | large VLDL | 6.11 [3.9 , 8.6] | 5.05 [3.0 , 7.2] |
| | | | | | | | P05 | large VLDL | 14.95 [11.7 , 19.3] | 15.64 [12.0 , 20.3] |
| | | | | | | | P06 | medium VLDL | 8.88 [7.3 , 11.6] | 8.75 [6.8 , 11.2] |
| | | | | | | | P07 | small VLDL | 6.56 [5.2 , 8.4] | 6.91 [5.1 , 9.0] |
| | | | | LDL | 84.41 [74.2 , 98.3] | 85.16 [76.5 , 102.7] | P08 | large LDL | 25.37 [20.8 , 30.0] | 26.83 [19.9 , 31.3] |
| | | | | | | | P09 | medium LDL | 37.90 [33.6 , 45.4] | 39.60 [34.5 , 48.2] |
| | | | | | | | P10 | small LDL | 14.17 [11.7 , 18.3] | 14.39 [11.0 , 19.6] |
| | | | | | | | P11 | very small LDL | 4.22 [3.6 , 5.4] | 4.46 [3.4 , 5.8] |
| | | | | | | | P12 | very small LDL | 0.30 [0.1 , 0.6] | 0.21 [0.1 , 0.6] |
| | | | | | | | P13 | very small LDL | 0.90 [0.8 , 1.0] | 0.87 [0.8 , 1.1] |
| | | | | HDL | 47.68 [41.3 , 59.5] | 50.46 [42.3 , 57.8] | P14 | very large HDL | 0.51 [0.4 , 0.7] | 0.58 [0.4 , 0.7] |
| | | | | | | | P15 | very large HDL | 2.03 [1.5 , 3.1] | 2.11 [1.6 , 3.0] |
| | | | | | | | P16 | large HDL | 8.79 [6.0 , 15.5] | 10.30 [5.7 , 14.2] |
| | | | | | | | P17 | medium HDL | 16.84 [14.2 , 21.5] | 18.48 [14.7 , 20.8] |
| | | | | | | | P18 | small HDL | 13.72 [11.8 , 16.1] | 13.34 [11.8 , 15.5] |
| | | | | | | | P19 | very small HDL | 3.74 [2.9 , 4.3] | 3.56 [2.8 , 3.9] |
| | | | | | | | P20 | very small HDL | 1.39 [1.3 , 1.5] | 1.37 [1.2 , 1.5] |

**52 week**

| | Anagliptin | Sitagliptin | P | 4 subclass | Anagliptin | Sitagliptin | Peak number of 20 subclass | Subclass name | Anagliptin | Sitagliptin |
|---|---|---|---|---|---|---|---|---|---|---|
| TC | 169.76 [159.5 , 192.4] | 181.88 [160.1 , 201.9] | 0.48 | CM | 5.45 [3.3 , 7.0] | 3.84 [2.1 , 7.6] | P01 | CM | 3.31 [1.9 , 4.8] | 2.20 [1.1 , 5.0] |
| | | | | | | | P02 | CM | 2.00 [1.4 , 2.5] | 1.51 [1.0 , 2.7] |
| | | | | VLDL | 41.11 [34.6 , 48.5] | 40.99 [32.6 , 48.3] | P03 | large VLDL | 4.42 [3.5 , 5.3] | 3.60 [3.1 , 5.4] |
| | | | | | | | P04 | large VLDL | 6.17 [5.1 , 7.2] | 4.93 [3.8 , 7.6] |
| | | | | | | | P05 | large VLDL | 14.12 [12.5 , 17.2] | 15.15 [12.2 , 19.6] |
| | | | | | | | P06 | medium VLDL | 9.14 [7.2 , 11.6] | 9.03 [7.6 , 10.9] |
| | | | | | | | P07 | small VLDL | 6.09 [5.1 , 7.0] | 5.54 [4.2 , 8.2] |
| | | | | LDL | 74.46 [68.0 , 93.5] | 84.31 [70.9 , 96.4] | P08 | large LDL | 24.25 [21.3 , 27.4] | 25.74 [19.6 , 31.7] |
| | | | | | | | P09 | medium LDL | 33.33 [30.3 , 43.5] | 37.38 [31.9 , 44.8] |
| | | | | | | | P10 | small LDL | 12.94 [10.0 , 17.6] | 13.34 [10.7 , 16.9] |
| | | | | | | | P11 | very small LDL | 3.71 [3.0 , 4.7] | 3.82 [3.2 , 4.7] |
| | | | | | | | P12 | very small LDL | 0.55 [0.4 , 0.7] | 0.61 [0.5 , 0.8] |
| | | | | | | | P13 | very small LDL | 0.79 [0.7 , 0.9] | 0.84 [0.8 , 1.0] |
| | | | | HDL | 48.74 [41.5 , 60.2] | 52.45 [44.2 , 59.5] | P14 | very large HDL | 0.62 [0.5 , 0.9] | 0.72 [0.6 , 0.9] |
| | | | | | | | P15 | very large HDL | 2.00 [1.5 , 2.7] | 2.15 [1.7 , 3.4] |
| | | | | | | | P16 | large HDL | 9.37 [7.0 , 14.9] | 11.30 [7.5 , 16.4] |
| | | | | | | | P17 | medium HDL | 17.46 [15.2 , 23.0] | 19.33 [16.1 , 21.5] |
| | | | | | | | P18 | small HDL | 13.03 [11.2 , 15.2] | 12.85 [11.3 , 14.6] |
| | | | | | | | P19 | very small HDL | 3.20 [2.8 , 3.8] | 3.45 [2.8 , 3.7] |
| | | | | | | | P20 | very small HDL | 1.38 [1.3 , 1.5] | 1.40 [1.3 , 1.5] |

*P < 005, **P < 001 between at 0 and 52 week.

TC: total cholesterol, TG: triglycerides, CM: chylomicron, CM-C: CM cholesterol, VLDL: very low-density lipoprotein, VLDL-C: VLDL cholesterol, VLDL: low-density lipoprotein, LDL-C: LDL cholesterol, HDL: high-density lipoprotein, HDL-C: LDL cholesterol,
