# Supplementary material for: Dissimilar Effects of Anagliptin and Sitagliptin on Lipoprotein Subclass in Standard or Strong Statin-Treated Patients with Type-2 Diabetes Mellitus: A Subanalysis of the REASON (Randomized Evaluation of Anagliptin versus Sitagliptin on Low-Density LipoproteiN Cholesterol in Diabetes) Trial
Source: J Clin Med. 2019 Dec 30;9(1):93. doi: 10.3390/jcm9010093 (PMC7019317; doi:10.3390/jcm9010093)
Supplement: Supplementary file 1 [file jcm-09-00093-s001.zip › jcm-657904-supplementary/Hirai_Supplement_2.pdf]

Supplement 2. Values of triglycerides in lipoprotein and lipoprotein subclass in patients treated either with anagliptin or sitagliptin at 0 and 52 week

0 week

	Anagliptin	Sitagliptin	P	4 subclass	Anagliptin	Sitagliptin	Peak number of 20 subclass	Subclass name	Anagliptin	Sitagliptin
TG	144.75 [108.5 , 187.7]	123.49 [90.0 , 174.2]	0.18	CM	14.14 [6.8 , 25.8]	10.00 [3.7 , 19.8]	P01	CM	8.30 [3.5 , 15.0]	5.62 [2.0 , 13.2]
							P02	CM	6.19 [3.0 , 10.0]	4.54 [1.7 , 7.7]
				VLDL	89.72 [61.3 , 123.1]	76.35 [43.8 , 103.9]	P03	large VLDL	15.03 [8.5 , 20.5]	11.07 [4.9 , 18.6]
							P04	large VLDL	24.47 [13.3 , 34.1]	17.78 [9.4 , 30.1]
							P05	large VLDL	28.79 [20.1 , 37.7]	23.65 [15.1 , 31.6]
							P06	medium VLDL	13.99 [11.3 , 17.8]	11.34 [8.9 , 17.4]
							P07	small VLDL	6.40 [5.4 , 7.5]	6.19 [5.0 , 7.8]
				LDL	25.17 [21.8 , 29.1]	25.75 [21.9 , 32.1]	P08	large LDL	9.89 [8.4 , 11.3]	10.24 [8.3 , 13.3]
							P09	medium LDL	9.48 [8.2 , 11.2]	10.32 [8.4 , 12.2]
							P10	small LDL	3.65 [3.1 , 4.5]	3.78 [3.0 , 4.9]
							P11	very small LDL	1.27 [1.1 , 1.6]	1.24 [1.0 , 1.5]
							P12	very small LDL	0.30 [0.2 , 0.5]	0.25 [0.1 , 0.4]
							P13	very small LDL	0.37 [0.3 , 0.5]	0.30 [0.3 , 0.4]
				HDL	15.57 [12.2 , 20.7]	14.70 [11.6 , 18.7]	P14	very large HDL	0.29 [0.2 , 0.4]	0.23 [0.2 , 0.4]
							P15	very large HDL	0.75 [0.5 , 1.3]	0.78 [0.5 , 1.1]
							P16	large HDL	3.46 [2.5 , 5.5]	3.73 [2.3 , 4.9]
							P17	medium HDL	5.38 [4.2 , 7.2]	5.20 [3.7 , 6.9]
							P18	small HDL	3.55 [2.9 , 4.7]	3.20 [2.3 , 4.5]
							P19	very small HDL	0.80 [0.5 , 1.1]	0.62 [0.4 , 0.9]
							P20	very small HDL	1.17 [1.0 , 1.4]	1.08 [1.0 , 1.3]

52 week

	Anagliptin	Sitagliptin	P	4 subclass	Anagliptin	Sitagliptin	Peak number of 20 subclass	Subclass name	Anagliptin	Sitagliptin
TG	120.47 [96.1 , 146.7]	103.23 [82.6 , 150.9]	0.56	CM	13.72 [8.8 , 23.0]	10.33 [5.4 , 22.6]	P01	CM	8.12 [4.8 , 15.8]	6.31 [3.0 , 14.3]
							P02	CM	5.20 [3.9 , 7.8]	4.00 [2.3 , 7.6]
				VLDL	66.20 [50.7 , 76.2]	51.72 [37.9 , 80.1]	P03	large VLDL	11.17 [8.1 , 13.9]	8.51 [5.3 , 14.0]
							P04	large VLDL	16.15 [11.3 , 20.0]	11.56 [8.0 , 19.9]
							P05	large VLDL	20.52 [15.3 , 23.6]	16.04 [12.4 , 24.7]
							P06	medium VLDL	11.24 [8.8 , 14.0]	9.77 [7.1 , 13.6]
							P07	small VLDL	5.32 [4.3 , 6.2]	5.08 [3.6 , 6.6]
				LDL	25.10 [21.0 , 28.4]	25.58 [22.3 , 30.5]	P08	large LDL	9.23 [7.8 , 10.7]	9.44 [8.0 , 11.4]
							P09	medium LDL	9.50 [8.0 , 11.5]	9.89 [8.9 , 12.0]
							P10	small LDL	3.83 [3.1 , 5.0]	3.99 [3.2 , 4.9]
							P11	very small LDL	1.20 [1.0 , 1.5]	1.21 [0.9 , 1.6]
							P12	very small LDL	0.44 [0.3 , 0.6]	0.40 [0.3 , 0.7]
							P13	very small LDL	0.35 [0.3 , 0.5]	0.34 [0.3 , 0.5]
				HDL	16.36 [14.0 , 21.3]	15.43 [12.2 , 19.8]	P14	very large HDL	0.36 [0.3 , 0.5]	0.31 [0.2 , 0.5]
							P15	very large HDL	0.78 [0.6 , 1.1]	0.86 [0.5 , 1.4]
							P16	large HDL	3.73 [3.1 , 5.5]	4.01 [2.8 , 5.8]
							P17	medium HDL	5.59 [4.6 , 7.4]	5.28 [4.0 , 6.8]
							P18	small HDL	3.63 [3.0 , 4.2]	3.08 [2.4 , 4.1]
							P19	very small HDL	0.71 [0.6 , 0.9]	0.65 [0.4 , 0.9]
							P20	very small HDL	1.23 [1.1 , 1.4]	1.18 [1.0 , 1.4]

*P < 005, **P < 001 between at 0 and 52 week.

TC: total cholesterol, TG: triglycerides, CM: chylomicron, CM-C: CM cholesterol, VLDL: very low-density lipoprotein, VLDL-C: VLDL cholesterol, VLDL: low-density lipoprotein, LDL-C: LDL cholesterol, HDL: high-density lipoprotein, HDL-C: LDL cholesterol,
